# Biochemical Prospects of Various Microbial Pectinase and Pectin: An Approachable Concept in Pharmaceutical Bioprocessing

**DOI:** 10.3389/fnut.2020.00117

**Published:** 2020-08-06

**Authors:** Sonali Satapathy, Jyoti Ranjan Rout, Rout George Kerry, Hrudayanath Thatoi, Santi Lata Sahoo

**Affiliations:** ^1^Microbiology Research Laboratory, Post Graduate Department of Botany, Utkal University, Bhubaneswar, India; ^2^School of Biological Sciences, AIPH University, Bhubaneswar, India; ^3^Post Graduate Department of Biotechnology, Utkal University, Bhubaneswar, India; ^4^Department of Biotechnology, North Orissa University, Baripada, India

**Keywords:** biochemical properties, depolymerization, pectic substances, pectinase, pharmaceutical application, production method

## Abstract

Both pectin and pectinase are vitally imperative biomolecules in the biotechnological sector. These molecules are a feasible non-toxic contrivance of nature with extensive applicative perception. Understanding pectic substances and their structure, unique depolymerization, and biochemical properties such as a catalytic mechanism and the strong interrelationship among these molecules could immensely enhance their applicability in industries. For instance, gaining knowledge with respect to the versatile molecular heterogeneity of the compounds could be considered as the center of concern to resolve the industrial issues from multiple aspects. In the present review, an effort has been made to orchestrate the fundamental information related to structure, depolymerization characteristics, and classification of pectin as well as the types and biochemical properties of pectinase. Furthermore, various production methods related to the optimization of the product and its significant contribution to the pharmaceutical industry (either pectinase or derived pectic substances) are described in this article.

## Introduction

Over the past centuries, there has been a notable increase in the application of enzymes in various sectors of industry. This is because the enzymes could be used as a substitute for some toxic chemicals that were previously involved in food processing. On the other hand, the biotechnological approaches involving the identification of microbial enzymes, mechanisms of action, and scaled-up production are a critical challenge to the researchers (1, 2). Protein engineering, in addition to recombinant processes, is an approachable alternative to engineer recombinant enzymes that enhance the rate of action in various aspects. There have been number of microbe-borne enzymes (amylase, cellulase, glucosidases, invertase, keratinases, lactase, ligninase, lipase, penicillinase, protease, xylanase, etc.) developed and commercially popularized due to their highly significant action as well as economic feasibility (3, 4).

The enzyme pectinase has engrossed interest worldwide as a biological catalyst in various industrial processes. This enzyme breaks down pectin commonly found in the plant cell wall and, hence, is well-known for the commercial preparation of clear fruit juice, liquefaction and saccharification of plant biomass, paper making, as well as coffee and tea fermentation (5). Pectin is a structural acidic heteropolysaccharide rich in galacturonic acid with carboxyl groups esterified with the methanol. The acidic heteropolysaccharide is a major component in cereals, vegetables, and fruits. Pectic substances are high in molecular weight, biocompatible, non-toxic, anionic natural polysaccharides, and foremost constituents of the middle lamella and primary cell wall of plants (6). Pectinase possesses a complicated enzymatic system responsible for the degradation of pectic substances like pectinic acids, propectins, pectins, and pectic acids (7). According to the enzymatic nomenclature system, pectinase hydrolyses the pectin polymer more efficiently than other pectinase groups of enzymes. Moreover, their actions are also more specific as they belong to a heterogeneous group of enzymes named as polygalcturonase (PG), pectinesterase (PE), and pectin lyase (PL) (8). The primary chain of pectin partly comprises methyl esterified 1,4-D-galacturonan and a demethylated form of pectin which is also known as pectic acid (pectate) or polygalacturonic acid. Basically, the pectinase acts by splitting glycosidic linkages and converts the polygalacturonic acid into monogalacturonic acid (9).

Microorganisms and plants have a proven record of being the major sources of the pectinase enzyme. However, because of technical and commercial viability, pectinase generation from microbial sources is becoming a primary area of research interest (9, 10). Naturally, this enzyme plays a significant role in the metabolic activities of almost all living organisms right from the complex to very simple ones such as plants, animals, fungi, bacteria, and viruses (10). However, researchers are now focused on how to improve its efficacy with respect to various aspects by involving various biotechnological tools. Furthermore, scientists are also emphasizing enabling or modifying pectinase so that it could be of application in different bioprocessing industrial sectors (11, 12). Pectinolytic enzymes are employed in many industrial applications but are mainly utilized in food industries in specific operations such as the clarification of fruit juices and wines and the extraction of vegetable oils. They are also widely used in the processing and elimination of pectin, which is crucial in coffee and tea processing, macerating of plants and vegetable tissue, degumming of plant fibers, treatment of wastewater, bleaching of paper, and as an additive in the poultry feeding and textile industries (13, 14). The benefaction of pectinase is also distributed toward maintaining intestinal microbiota homeostatic balance by improving nutritional prebiotic values by processing plant-based foods (15). Enzymatic preparations of pectin are also implemented in various pharma-based industries for the development of low-methoxy pectin that is beneficial for diabetic patients (14, 16). Purified forms of pectinase, which are commercially isolated from different fungal species, have a great role in plant protoplast culture studies as they help to generate good yields of viable protoplast when treated with cellulose (17). However, like other enzymes, this one is also more activated during its commercial production at 30 to 50°C and a pH of 4.5 to 8.5, but the effectiveness is more specific to the source of particular microorganisms and other environmental factors (10).

Hence, due to market and economic demand, the detailed information about the structure, depolymerization properties, and classification of pectic substances as well as the types and biochemical properties of pectinase are collected from literature search and presented in the present review. In addition, the review also presents an overall idea about the different production methods of pectinase and pharmaceutical applications of both pectin and pectinolytic enzyme. Thus, it is hoped that the review article may support the building of conceptual knowledge on the protease enzyme and its substrate.

## Production Methods of Selected Pectinase From Microorganisms

Naturally occurring pectinases are basically found in either plants or microorganisms, but their isolation and commercial production from microbial sources will meet the industrial needs through a large-scale production involving the fermentation process. The initial isolation and identification of selective microbes, screening of selective media, and production process either via flask culture or scale-up culture (fermentation process) are the critical tasks to optimize the individual enzymes. Moreover, the standardization of the substrate concentration optimization with respect to environmental factors is also a critical factor to postulate an effective function of pectinolytic enzymes. The significant contribution of these enzymes has drawn considerable attention from different biotechnologists worldwide to enhance their potentiality by adapting several recombinant strategies (18, 19). The commercialized pectinase enzymes are produced by either submerged fermentation (SmF) or solid-state fermentation (SSF) that varies in the use of selected fungus or bacteria (20–22). The SmF technique has been adopted since 1940s when the microorganisms were cultivated on liquid broth with constant agitation for the large-scale production of antibiotics and later widely applied in various industries for the production of a huge assortment of microbial metabolites (23, 24). On the other hand, the SSF is also applicable in various sectors for enzyme production where microorganisms are cultured on solid materials which are eco-friendly, cost-effective, and highly productive and have high biomass yield, dwindled proteolytic impact, higher resistance to catabolic repression, better product quality, etc. (25, 26). The selection or adaptation of a specific technique as well as a suitable medium depends on selected microorganisms, including some surrounding environmental factors for the optimization of pectinase production. Some significant contributions relating to selectable substrate-containing media and their production methods are listed in Table 1.

**Table 1:** List of various screening or production media and production processes for the discovery of specific pectinase enzymes.

**Enzymes**	**Enzyme subtypes**	**Source/microorganism**	**Screening/production media [clue additive(s)]**	**Production process**	**References**
Protopectinase		*Aspergillus awamori* IF0 4033	Wheat bran with 0.2 M HCl	SSF	(27)
		*Geotrichum klebahnii* ATCC 42397	Synthetic media	SmF	(28)
		*Aspergillus terreus*	Sucrose with yeast extract, meat peptone, K_2_HPO_4_, KH_2_PO_4_, and MgSO_4_	SmF	(29)
		*Paenibacillus polymyxa* Z6	Apple pomace, peptone, K_2_HPO_4_, MgSO_4_, and CaCl_2_	SmF	(30)
Esterase	Pectin methylesterase	*Aspergillus niger*	Dried apple pomace powder	SSF, SmF	(31)
		*Aspergillus heteromorphus*	Pectin agar	SmF	(32)
		*Penicillium notatum* NCIM. 923	Wheat bran and dried orange peel	SSF	(33)
		*Fusarium asiaticum*	Liquid cultures with potato tuber extract	Gel diffusion	(34)
		*Cladosporium cladosporioides*	Semi solid rice	SmF	(35)
		*Aspergillus tubingensis*	Dried papaya peel	SSF	(36)
		*Aspergillus niger*	Wheat bran, citric pectin, salt solution	SSF	(20)
		*Streptomyces coelicoflavus* GIAL86	Pectin, yeast extract, bacteriological agar	SmF	(37)
	Pectin acetylesterase^*^	*Erwinia chrysanthemi* 3937	Luria-Bertani/synthetic M63	SmF	(38)
Depolymerase	Polygalacturonase	*Neurospora crassa*	Vogel's minimal supplemented with 2% (w/v) glucose	SmF	(39)
		*Aspergillus japonicas* 586	Manachini solution	SmF	(40)
		*Bacillus* sp.	Wheat bran, 1% (NH_4_)_2_SO_4_, 0.02% MgSO_4_ solution	SSF	(41)
		*Kluyveromyces marxianus* CCT 3172	Semi synthetic	SmF	(42)
		*Aspergillus awamori*	Grape pomace	SSF	(43)
		*Aspergillus fumigatus* Fres. MTCC 4163	Yeast soluble starch agar with pectin	SSF	(44)
		*Saccharomyces* sp.	YPD (Glucose 2%, yeast extract 1%, peptone 1%) YPGal (Galactose 2%, yeast extract 1%, peptone 1%)	SmF	(45)
		*Bacillus firmus* –I-4071	Basal medium supplemented with potato peels, *Echornia crassips*, and citrus peels mixture	SSF	(46)
		*Aspergillus giganteus*	Vogel medium containing ammonium sulfate source and orange bagasse	SmF	(47)
		*Aspergillus niger*	Czapek-Dox agar with pectin	SmF	(48)
		*Aspergillus* strains	Pectinase screening agar medium	SSF SmF	(49)
		*Penicillium chrysogenum*	Yeast soluble starch agar with pectin	SmF	(50)
		*Penicillium* sp.	Pectin agar	SSF SmF	(51)
		*Mucor circinelloides* ITCC6025	KCl, MgSO_4_.7H_2_O, trisodium citrate dihydrate, citric acid anhydrous, yeast extract, casein hydrolysate, pectin	SmF	(52)
		*Bacillus* sp.	Yeast extract pectin medium	SmF Semi Solid Fermentation	(53)
		*Bacillus cereus*	Pectate agar plates amended with polygalacturonic acid	SmF	(54)
		*Paecilomyces marquandii* *Aspergillus fumigatus* *Trichosporiella cerebriformis* *Mortierella* sp. *Syncephalastrum recemosum*	Pectin agar	SmF	(55)
		*Bacillus* sp.	Yeast extract pectin calcium chloride medium	SmF	(56)
		*Aspergillus fumigatus*	Mango pectin, NH_4_NO_3_, NH_4_ H_2_PO_4_, MgSO_4_.7H_2_O	SmF	(57)
		*Aspergillus foetidus*	Dried mango peel powder with salt solution	SSF SmF	(58)
		*Aspergillus niger*	Sabouraud dextrose agar supplemented with pectin	SmF	(59)
		*Rhizomucor pusillus*	Yeast soluble starch agar supplemented with pectin	SSF	(60)
		*Aspergillus flavus, Aspergillus niger, Aspergillus ochraceous*	Pectin agar having 1% pectin	SmF	(61)
		*Cladosporium cladosporioides*	Semi solid rice	SmF	(35)
		*Aspergillus niger* HFD5A-1	KH_2_PO_4_, Na_2_HPO_4_, FeSO_4_.7H_2_O, CaCl_2_, (NH_4_)_2_SO_4_, MnSO_4_.7H_2_O, H_3_BO_3_, citrus pectin	SmF	(62)
		*Aspergillus* sp.	Liquid culture medium consists of orange peels	SmF	(63)
		*Wickerhamomyces anomalus*	Yeast nitrogen base agar	SmF	(64)
		*Bacillus* sp. *Erwinia* sp.	Mc Beth's medium	SmF	(65)
		*Streptococcus* sp. *Staphylococcus aureus* subsp. *anaerobius*	Standard growth medium containing pectin	SmF	(66)
		*A. sojae* ATCC 20235	Orange peel, Maltrin or glucose	SmF	(67)
		*Penicillium canescens* *Rhizopus stolonifer* *Aspergillus candidus* *Gliocladium viride* *Penicillium* sp.	Czapek-Dox agar supplemented with pectin and congo red	SmF	(68)
		*Bacillus licheniformis*	Pectin agar	SmF	(69)
		*Penicillium lividum*	Czapek-Dox agar with pectin	SmF	(70)
		*Aspergillus oryzae, Aspergillus flavus, Rhizopus oryzae*	Czapek-Dox agar with commercial citrus pectin	SSF SmF	(71)
		*Aspergillus niger* *Aspergillus fumigatus* *Aspergillus flavus*	NH_4_NO_3_, NH_4_H_2_PO_4_, MgSO_4_.7H_2_O, ground mango peel	SmF	(72)
		*Aspergillus niger* MTCC 281	Dried banana peel powder with salt solution	SmF	(73)
		*Staphylococcus aureus, Bacillus cereus*	Minimal essential agar medium with 2% pectin	Kirby Bauer disc diffusion	(74)
		*Aspergillus niger* MCAS2	Potato dextrose broth supplemented with 0.5% pectin	SmF	(75)
		*Aspergillus niger*	Czapek- Dox supplemented with orange waste peel	SmF	(76)
		*Aspergillus oryzae*	KH_2_PO_4_, K_2_HPO_4_, MgSO_4_.7H_2_O, (NH_4_)_2_SO_4_, yeast extract	SmF	(77)
		*Bacillus subtilis*	Pectinase screening agar medium	SmF	(78)
		*Aspergillus niger* MTCC 478	Solid pectin agar	SSF SmF	(79)
		*Bacillus licheniformis* KIBE-IB3	Pectin agar	SmF	(80)
		*Aspergillus niger* IBT-7	Pectinase screening agar	SSF	(81)
		*Aspergillus niger*	Pectinase screening agar	SmF	(82)
		*Aspergillus oryzae*	Pectinase screening agar containing 1% of pectin	SSF	(83)
		*Aspergillus oryzae* RR103	Pectin agar	SmF	(84)
		*Bacillus subtilis* strain NBT-15	Pectinase screening agar	SmF	(18)
		*Chryseobacterium indologenes*	Yeast extract pectin agar	SmF	(85)
		*Tetracladium* sp.	Yeast malt medium supplemented with glucose	SmF	(86)
		*Bacillus* sp. Y1	Wheat bran, starch, (NH_4_)_2_SO_4_, MgSO_4_·7H_2_O, Na_2_CO_3_, Tween80	SmF	(87)
		*Bacillus licheniformis* UNP-1	Pectin agar (Pectin, KH_2_PO_4_, K_2_HPO_4_, MgSO_4_.7H_2_O)	SmF	(88)
		*Bacillus tequilensis* SALBT	Pectin agar	SmF	(89)
		*Aspergillus fumigatus*	Pectin agar	SSF	(90)
		*Aspergillus niger* ATCC 120120	(NH_4_)SO_4_, MgSO_4_.7H_2_O, KH_2_PO_4_, FeSO_4_.7H_2_O, yeast extract, glucose and pectin of *Nephrolepis biserrata* leaves	SSF	(91)
		*Aspergillus* sp.	Czapek-Dox agar with citrus pectin	SmF	(92)
		*Streptomyces coelicoflavus* GIAL86	Pectin, yeast extract, bacteriological agar	SmF	(37)
		*Bacillus tequilensis* CAS-MEI-2-33	Pectin agar	SmF	(93)
		*Geotrichum candidum AA15*	Mineral salt medium	SmF	(94)
		*Penicillium janczewskii*	Wheat bran	SSF	(95)
		*Bacillus amyloliquefaciens SW106* *Bacillus subtilis TYg4-3*	Pectinase screening agar	SmF	(96)
		*Penicillium chrysogenum*	Beet pulp powder medium	SmF	(97)
		*Bacillus megaterium*	Pectin agar medium	SmF	(22)
		*Aspergillus niger*	Minimal media (pectinase screening agar medium)	SmF	(98)
		*Alternaria solani, Curvularia lunata, Geotrichum candidum, Rhizoctona solani, Fusarium oxysporum, Phytophthora* sp.	Pectin, KNO_3_, KH_2_PO_4_, MgSO_4_. 7H_2_O, amino acids in 0.02% concentration	SmF	(99)
		*Alternaria solani, Geotrichum candidum, Aspergillus niger, Aspergillus flavus, Penicillium expansum*	Glucose nitrate, pectin peptone, pectin nitrate, and pectin ammonium nitrate individually	SmF	(100)
		*Aspergillus fumigates MS16*	Mineral salt medium supplemented with grounded banana peels	SSF, SmF	(101)
		*Neurospora crassa*	Vogel's minimal medium supplemented with 2% (w/v) glucose	SmF	(39)
	Lyase	*Penicillium chrysogenum*	Solid pectin agar medium	SmF	(102)
		*Penicillium griseoroseum*	Mineral medium supplemented with yeast extract and sucrose	SmF	(103)
		*Saccharomyces* sp.	YPD (Glucose 2%, yeast extract 1%, peptone 1%) YPGal (Galactose 2%, yeast extract 1%, peptone 1%)	SmF	(45)
		*Aspergillus foetidus*	Dried mango peel powder with salt solution	SSF SmF	(58)
		*Aspergillus oryzae*	Pectinase screening agar medium plates containing 1% of pectin	SSF	(83)
		*Aspergillus niger*	Wheat bran, citric pectin, salt solution	SSF	(20)
		*Schizophyllum commune*	Mosambi (sweet lime) fruit peels	SSF	(104)
		*Streptomyces coelicoflavus* GIAL86	Pectin, yeast extract, bacteriological agar	SmF	(37)
		*Escherichia coli* BL21	Glycine added medium	SmF	(105)
					

* *Limited literature; SSF, Solid-state fermentation; SmF, Submerged fermentation*.

## Classification and Biochemical Properties of Pectinolytic Enzymes

**Figure 1:**
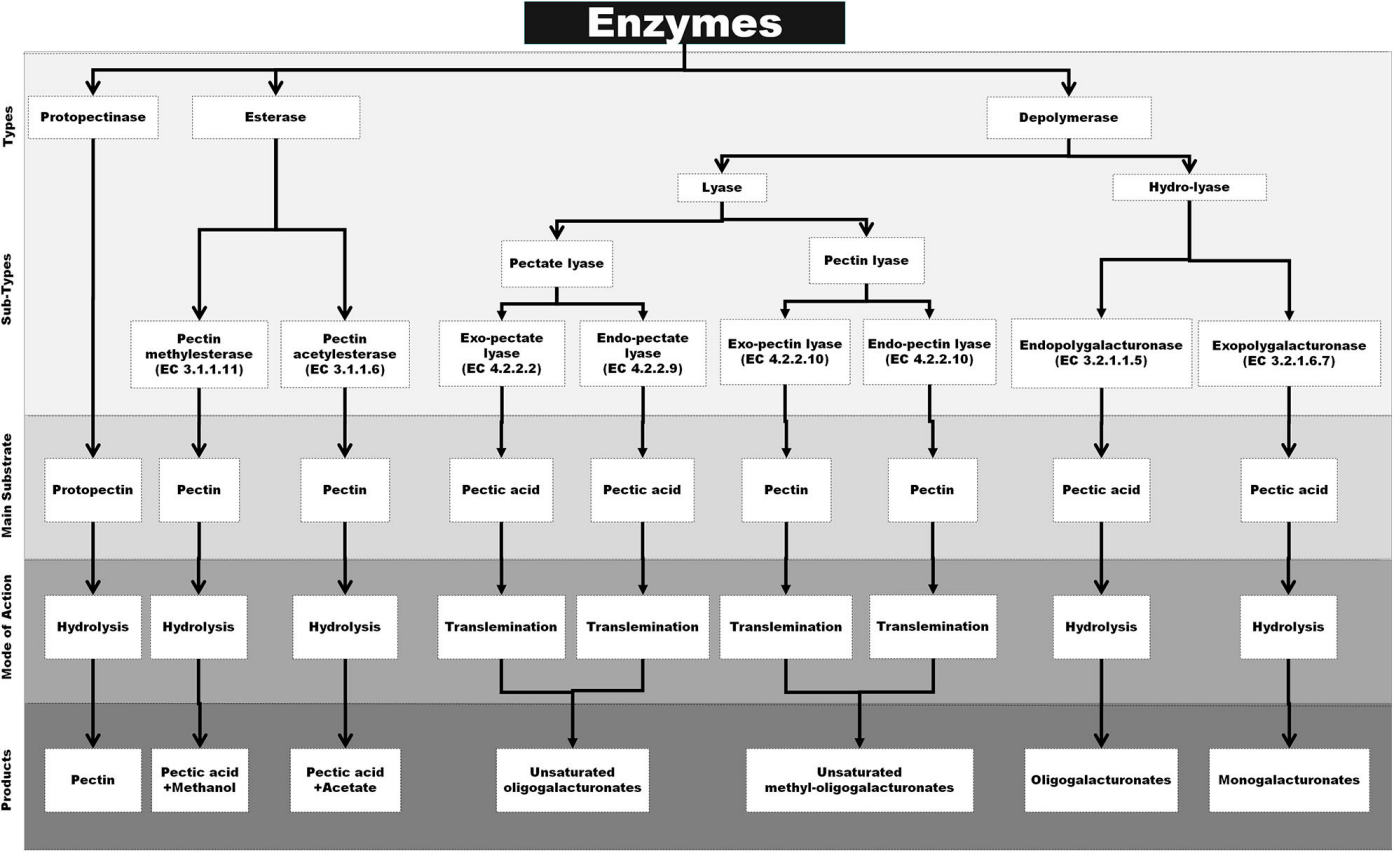
Schematical representation of specific substrate, mode of action and end products of various pectinase [Inspired from Garg and Singh (107)].

Pectic enzymes are also known as pectinases or pectinolytic enzymes, which have the capacity to hydrolyze the various complex pectic substances. These enzymes are classified into three major types including protopectinases, esterase, and depolymerases, on the basis of their mechanism of action on pectin molecules and preferred substrates. *Protopectinases* is a group of enzymes that are responsible for the hydrolysis of the substrate protopectin and converting it to soluble pectin. *Esterase* is a class of enzymes that removes methoxyl and acetyl esters from pectin resulting in the formation of polygalacturonic acid. *Depolymerases* also contribute toward the breakdown of peptic substances by the cleaving of α-(1 → 4)-glycosidic bonds in D-GalA units either by hydrolysis or by trans-elimination (13, 106). However, the above system of classification is outdated, and most recently, the pectic enzymes are broadly separated as per the nature of action mechanism, primary substrate, and products that are narrated below and also schematically outlined in Figure 1.

### Protopectinase

Protopectinases (PPases) are also synonymous to pectinosinases, which interact with insoluble protopectin in the presence of water and converts it into simple and soluble pectin. The term was initially used around 1927 and recently popularized due to its importance in various sectors of industry like the production of pectin, single-cell protein, and protoplast (108, 109). More specifically, these enzymes could catalyze the protopectin by reacting at sites having three or more non-methylated GalA molecules and hydrolyzing the glycosidic bond (106, 110). Based on the catalytic action, these are categorized as type A PPase, which reacts with the polygalacturonic acid region or inner site of insoluble protopectin, whereas type B PPase responds at the site of polysaccharide chains or outside of the insoluble protopectin, which links the polygalacturonic acid chain and the cell wall components (110).

Type A PPase has also been reported in the culture filtrate of many yeasts like fungi such as *Kluyveromyces fragilis* IFO 0288, *Galactomyces reessi*, and *Trichosporon penicillatum* SNO 3, referred to as PPase-F, -L, and -S, respectively. The biological properties of all the three types of PPases are alike, possessing the same molecular weight of about 30 kDa. Further, it was found that all three types of enzymes were more optimum at pH 5.0. Among the three A types of PPases, only PPase-F is an acidic protein, while others are basic in properties. These enzymes play a vital role in the hydrolysis of polygalacturonic acid and also responsible for a decrease in viscosity with an enhanced rate of reduction in a certain reaction medium containing polygalacturonic acid. Similarly, PPase-N and -R were also identified from *Bacillus subtilis* IFO 3134 with 43 and 35 kDa protein, respectively, which help in transelimination reaction by splitting the glycosidic linkages at the region of protopectin. The enzymes were highly active at the same pH (8.0) and temperature (60°C) (111, 112). Type B PPases have been isolated from the pure culture of *B. subtilis* IFO 3134 and *Trametes sunginea*, which are also named as PPase-C and -T, respectively. In this case, different molecular weights of proteins like 30 (PPase-C) and 55 kDa (PPase-T) were detected along with different isoelectric points (PPas-C: 9.0; PPase-T: 8.1). As reported, these types of enzymes are abundantly found in agro-products like lemon, orange, hassaku, apple, burdock, carrot, radish, sugar beet, etc. where they particularly act on protopectin (109, 113).

### Esterase

Pectinesterases are the enzymes which help to separate methoxyl and acetyl residues from pectin, resulting in the formation of polygalacturonic acid. Pectinesterases from fungal sources work in a multi-chain mechanism by eliminating the methyl groups but in an arbitrary manner, whereas the pectinesterases from plant sources work in a single-chain mechanism, by targeting either the non-reducing terminal or that next to a free carboxyl group and then advancing in a linear fashion (114, 115). Pectinesterases are classified into two types as per the targeting functional groups, which are named as pectin methylesterase or pectinesterase and pectin acetylesterase. Pectin methylesterase works in single-chain mechanism by dividing the methyl ester group of pectin and thereby freeing methanol and converting pectin into pectate; but in this action, the length of the pectic chains are not reduced (116). Variably, the enzyme pectin acetylesterase catalyzes the hydrolysis of acetyl ester residues of pectin, resulting in pectic acid and acetate formation (110).

The screening and production of this category of enzymes have been done in maximum from microbiological sources as compared to plants and animals (112, 117). The rate of enzymatic action of pectinesterase on the viscosity of a solution containing pectin is almost negligible without the divalent cations like calcium (Ca^2+^), barium (Ba^2+^), and strontium (Sr^2+^). However, the maximum and effective involvement of Ca^2+^ ions is reported as either small- or large-scale preparations (118). Basically, some of the purified pectinesterases are targeted to the reducing end, whereas others are acting against the non-reducing end of the pectin. As per molecular characterization, most of the enzymes ranging from 22 to 90 kDa indicate a diversification of protein confirmations. However, these variations are organism-specific. Their work efficiency also varies with different pH levels and temperatures and ranges in pH from 5 to 11 and temperature from 40 to 70°C. However, it was depicted that fungal pectinesterases have a lower pH level for the optimization of products as compared to that of bacterial pectinesterases (13). The separation of various unique isoenzymes of pectin methylesterases have been achieved and characterized from different sources with respect to targeted functional groups (116, 119). The structural characterization of two pectin methylesterases such as PmeA and PmeB are well-studied from *Erwinia chrysanthemi* (120) and *Erwinia chrysanthemi* 3937 (121) and functionally found to be extracellular and outer membrane acting enzymes, respectively.

### Depolymerase

#### Polygalacturonase

Among all pectinolytic enzymes, polygalacturonases (PGases) are the most studied and industrially applied enzymes due to their depolymerization specificity through the hydrolysis process. These enzymes are specifically interactive through hydrolysis and split the glycosidic linkage in the presence of water molecules across the oxygen bridge. Polygalacturonase loses its structural confirmation when it reacts with pectin, which may occur because of the target molecules of polygalacturonases that have free carboxylic groups. During interaction with substrates, the viscosity of the solution reduces to a greater extent with an increase of reducing end groups (112). Polygalacturonases are classified into three types (exopolygalacturonase, endopolygalacturonase, and rhamnopolygalacturonase) depending on their pattern of action. Exopolygalacturonase targets the terminal groups of the pectic molecule, which results in gradual lessening of chain length, whereas endopolygalacturonase attacks arbitrarily on all the chain links, which results in faster and more incisive consequences. However, rhamnopolygalacturonase catalyzes haphazardly within or at the non-reducing terminals of rhamnogalacturonan core chains (122, 123).

There are several important biochemical properties and modes of actions of polygalacturonases obtained from various microbial sources. Most of the polygalacturonase enzymes stimulate the rate of hydrolysis at an ideal pH ranging from 3.5 to 5.5 with a suitable temperature that ranges from 30 to 50°C. Several findings relating to various biochemical properties like molecular weight, pH, temperature, isoenzyme, isoelectric point, etc. are well-reported with respect to endopolygalacturonase in various bacterial and fungal species as compared to exopolygalacturonase and rhamnopolygalacturonase. As reported, almost all endopolygalacturonase as well as exopolygalacturonase enzymes are synthesized in acidic environmental conditions, whereas, some exopolygalacturonases are produced at high basic conditions (about pH 11.0) and by particular species including *Bacillus licheniformis, Bacillus* sp KSM-P410 and *Fusarium oxysporum* (13). Regarding rhamnopolygalacturonases, it was stated that the enzymes are more stable and efficiently work at pH 4.0 and a temperature of 50°C (124). At an average molecular weight of 38–65 kDa, the enzymes (for both exopolygalacturonases and endopolygalacturonases) are separated from various microbial sources (13). However, at a high molecular weight of 496 kDa, enzymes were also isolated from *Kluyveromyces marxianus* (125). Similarly, the protein having a molecular weight of 66 kDa is electrophoresed from the rhamnopolygalacturonase (124).

#### Lyase

Lyases (also known as transeliminases) act by performing the trans-eliminative breakdown of pectate or pectinate polymers. The lyases typically split the glycosidic linkages at 4th carbon followed by the removal of a hydrogen atom from 5th carbon that results in an unsaturated product. On the basis of the substrates acted upon, lyases are classified into two major types, i.e., polygalacturonate lyase and polymethylgalacturonate lyase. However, they are further subdivided into five subtypes, namely, endo-polygalacturonate lyase, exo-polygalacturonate lyase, endo-polymethylgalacturonate lyase, exo-polymethylgalacturonate lyase, and oligo-D-galactosiduronate lyase as per their pattern of action (115). Polygalacturonate lyases (or pectate lyases) are further divided into two types depending on the pattern of action, i.e., endo-polygalacturonate lyases that work on the substrate in an unsystematic fashion, whereas exo-polygalacturonate lyases target the substrates from non-reducing terminal of pectic acid. In the same way, polymethylgalacturonate lyases (or pectin lyases) are also divided into two categories such as endo-polymethylgalacturonate lyases (that randomly act on the substrate pectin by cleaving α-1,4-glycosidic linkages and producing unsaturated methyloligogalacturonates) and exo-polymethylgalacturonate lyases (that degrade pectin by trans-eliminative cleavage but stepwise and produce unsaturated methylmonogalacturonates) (126, 127). The oligo-D-galactosiduronate lyases actively participate in the trans-elimination reaction at the terminal position of unsaturated digalacturonate that is initially produced by the action of pectate lyases and converted into unsaturated monogalacturonates (128, 129).

Microorganisms are the good sources for isolation of pectin lyases, but the biochemical properties of each isolated lyase are different. Polygalacturonate lyases or pectate lyases exclusively require Ca^2+^ ions for its activation. However, ions like Co^2+^, Mn^2+^, and Ni^2+^ are needed for activation of some cytoplasmic or intracellular lyases (126). It is reported that the polymethylgalacturonate lyases or pectin lyases do not require any metal ions for their activation, but arginine residues are found at the Ca^2+^ ion position as observed in the case of pectate lyases (130). Overall, both types of enzymes are efficient for working in an alkaline pH range of 7.5–10.0 and a temperature between 40 and 50°C. The molecular weights of lyases are ranging from 22 to 90 kDa, whereas the molecular weights of polymethylgalacturonate lyases were found to be 89 and 90 kDa in *Aureobasidium pullulans* LV-10 and *Pichia pinus*, respectively. For polygalacturonate lyases, the molecular weights of 55 and 74 kDa of proteins were reported in *Yersinia enterocolitica* and *Bacteroides thetaiotaomicron*, respectively. The isoelectric point of some lyases range from 5.2 to 10.7; however, others still need to be explored (13).

## Structure and Depolymerization of Pectin

The naturally occurring pectins or pectic substances are chiefly composed of galacturonic acid (GalA)-rich polysaccharides in the form of covalently-linked structural motifs that include homogalacturonan (HG), xylogalacturonan (XGA), rhamnogalacturonan I (RG-I), and rhamnogalacturonan II (RG-II). However, the occurrences of HG are maximum (about 65%) in pectin (131, 132). About hundred GalA moieties are linked by α-D-1,4- bonds and form HG that is further modifiable by the process of methyl-esterification at C-6, or acetyl groups at O-2 and O-3. Similarly, XGA is an HG backbone, where 25–75% of the GalA units are substituted at C-3 with one xylose moiety and sometimes with a second xylose residue at C-4 (114, 132). RG-I is also a complex polymer which contributes 20–35% of pectin in recurring units of α-D-GalA-1 and 2-α-L-Rha-1-4-disaccharide. Sometimes, lateral chains contain fucose and glucuronic acids available mostly to create a more complex structure. RG-II is another structural domain of pectin where the GalA residue acts as the main chain bonded by α-D-1,4- bonds and substituted with a usual L-rhamnose or D-galactose and numerous atypical sugars and laterally conjugated by 12 different sugar molecules. Moreover, the pectin matrix properties are complicated due to the interaction of the structural domains of pectin between themselves and with other ionized organic and inorganic compounds (131–133).

Depolymerization of complex pectin and subsequent conversion into simpler forms, i.e., pectic oligosaccharides, is the center of attraction because these molecules are very large but less complex and heterogeneous in comparison to their parent compounds. Furthermore, the oligosaccharides are exploited for many vital applications like repressor of liver lipid accumulation, antioxidant and cancer cell proliferation inhibitor, anti-metastatic agent, angiogenesis inhibitor, antibacterial agent, and formation of prebiotics (134–137).

**Figure 2:**
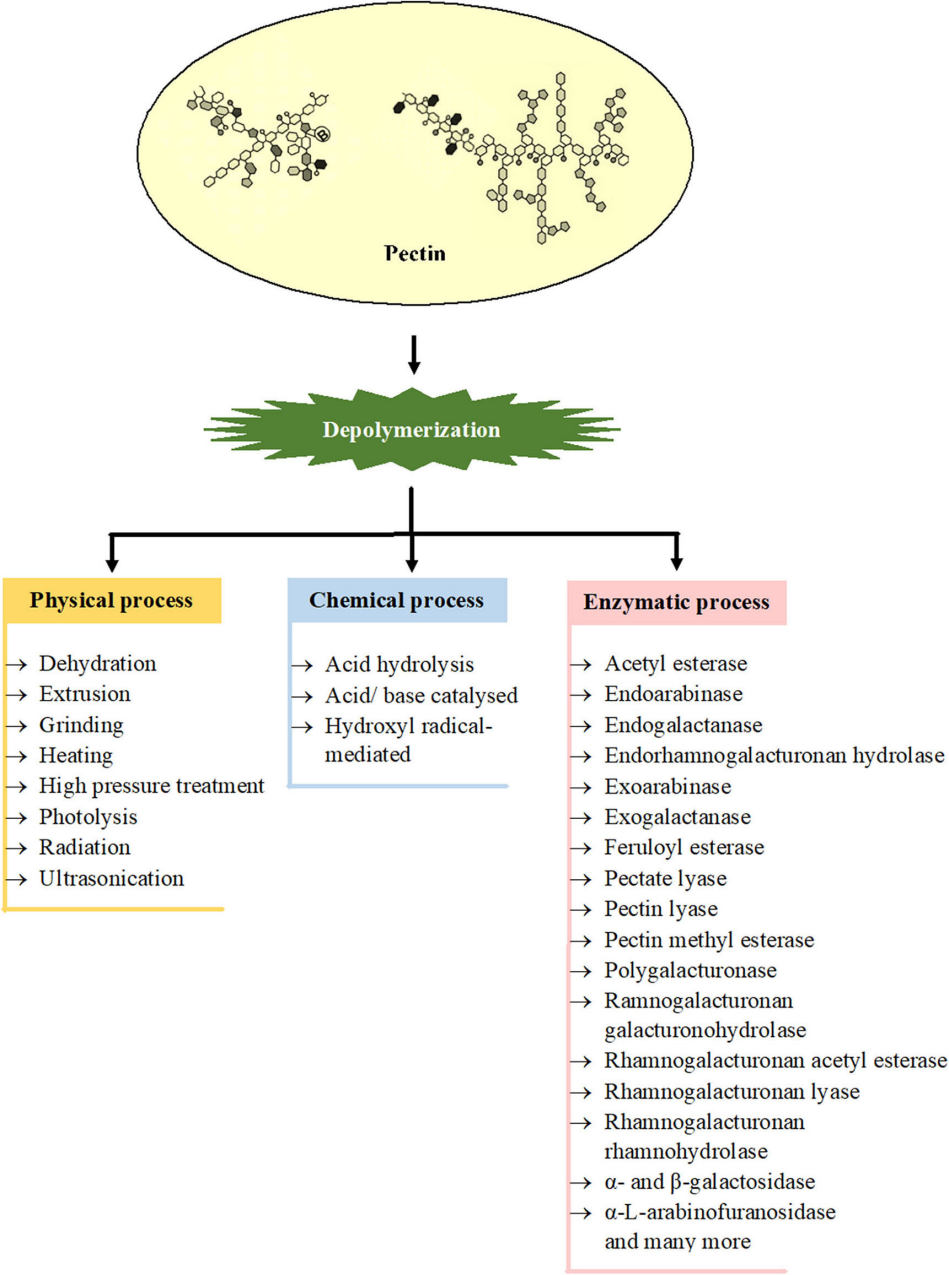
Involvement of different processes for depolymerization of various types of pectins [Inspired from Chen et al. (6)].

Degradation of pectin either by physical, chemical, or enzymatic methods is represented in Figure 2. Ultrasonication, high pressure treatment, radiation, and photolysis are some of the physical means used in degradation. In an aqueous solution of pH at about 3.5, the pectins are most stable, but at lower or higher pH, the methoxyl, acetyl, and neutral sugar groups are eliminated and the polymer backbone is cleaved. Chemically, the heteropolysaccharides can be degraded either by acid hydrolysis or base-catalyzed splitting of chains through the β-elimination reaction (138). The dissolution takes place at a glycosidic linkage next to an esterified GalA, as a result of which pectins with an elevated degree of methoxylation (DM) become extra susceptible to base-catalyzed reactions rather than pectin with a low DM. However, by acid hydrolysis (pH < 3.0), pectin hydrolyses with low DM are faster in comparison to pectin with high DM (139). Enzymatic degradation of pectin is gaining prime importance because it allows region-selective depolymerization under mild conditions. Due to the intricate molecular configuration of pectin, a wide range of enzymes are essential for the degradation of this polymer. These enzymes include polygalacturonase (PG) which degrades HG by hydrolyzing the glycosidic bonds and are categorized into two types, endo-PG and exo-PG (140). Lyases that include pectate lyase and pectin lyase catalyze the depolymerization of polygalacturonate and esterified pectin, respectively through β-elimination process in which a proton is removed and an unsaturated bond is formed between the C-4 and C-5 carbon atoms of the non-reducing terminal of pectin. PLYs in combination with Ca^2+^ degrade non-esterified pectin, and PEL in combination with Arg^236^ degrades methyl-esterified pectin (13). Some other residues of pectin such as acetyl, methyl, and feruloyl are separated by pectin methylesterase, acetyl esterase, rhamnogalacturonan acetyl esterase, and feruloyl esterase. The actions of these enzymes are imperative for the absolute degradation of pectin, as they enhance the action of other enzymes; for instance, the ability of PGs and PLYs to degrade pectin largely depends on the activity of PMEs (141, 142). The endorhamnogalacturonan hydrolase breaks -α-D-GalA-1,2-α-L-Rha linkages present in RG-I by hydrolysis, and rhamnogalacturonan lyase breaks -α-L-Rha-1,4-α-D-GalA linkages by β-elimination reaction. Accessory enzymes like rhamnogalacturonan rhamnohydrolase and ramnogalacturonan galacturonohydrolase break down oligosaccharides from the non-reducing terminal by an exo-attack (114). There are few more enzymes which also act on the adjacent chains of RG-I and RG-II such as endogalactanase, exogalactanase, α- and β-galactosidase, α-L-arabinofuranosidase, endoarabinase, and exoarabinase (143).

## Structure and Classification of Other Pectic Substances

**Figure 3:**
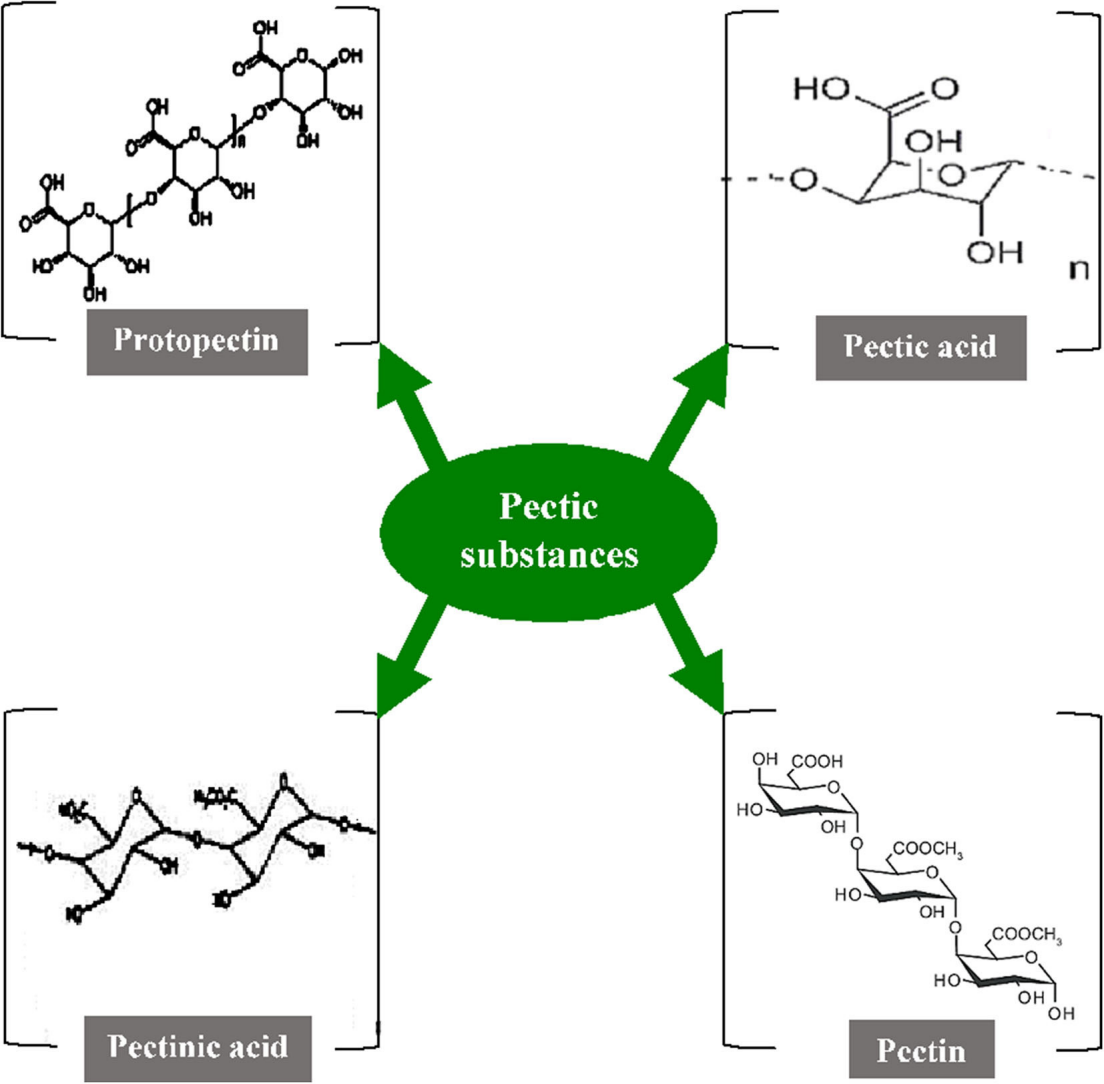
Structure of various pectic substances [Inspired from Garg and Singh (107)].

Pectic substances (including pectins) are complex heteropolysaccharides that contribute to the major elements of the middle lamellae and primary cell wall of higher plants. They play very diverse roles such as maintaining the integrity and coherence of plant tissues, acting as moisturizing or gluing agents in the cell walls, and also getting involved in the plant host-pathogen interaction (144, 145). In general, galacturonans and rhamnogalacturonans are two major chemical components in pectic substances, where the C-6 carbon of galactate is oxidized along with arabinans and arabinogalactans. The components produced are structurally heterogeneous and colloidal polymeric in nature, containing a large backbone of anhydrogalacturonic acid units (131). The carboxyl groups present in the GalA are partially esterified by methyl residues and moderately or totally neutralized by different ions such as sodium, potassium, and ammonium. The α-1,4-D-galacturonate units act as the principal chain and link about 2–4% of L-rhamnose units which are linked [β-(1 → 2) and β-(1 → 4)] to the galacturonate units. The side chains comprise simple carbohydrates that are arabinan, galactan, arabinogalactan, xylose, or fucose but vary in their composition and length. They are linked to the central chain through their C_1_ and C_2_ atoms (146). As per the nature of the molecular arrangements of pectic substances, the American Chemical Society have categorized them into four major groups namely protopectin, pectic acid, pectinic acid, and pectin (Figure 3) (147). The *protopectin* is a water-insoluble substance that acts as a cementing agent in the cell wall and, under hydrolysis, produces either pectin or pectic acid. Similarly, *pectic acid* (popularly known as pectate) is a polymer of galacturonans that contains a small number of methoxyl groups. *Pectinic acid* is a chain of polygaluturonates that mainly contains methylated galacturonate units. *Pectin* a polymeric substance with at least 75% of the carboxyl groups attached to the galacturonate units and is esterified with a methyl group (148).

## Extraction and Purification Methods of Pectin

The growing requirements of pectin in pharmaceutical applications have accelerated the need for competent extraction processes. Commercially, these naturally occurring biomolecules can be extracted from the peels of citrus fruits by alcohol precipitation (149). In the extraction process, the recovery of pectins is vital at the industrial scale to endow with passable supply for the increasing need. At the commercial level, the hydrolyzation of proto pectin into pectin at elevated temperature using acid and subsequently precipitating by ethanol is conventionally employed (150). Limitations like lower yield, heterogenic changes in macromolecular and gelling properties, etc. have raised the urgency to find novel methods like microwave-assisted extraction, enzymatic extraction (polygalacturonase, hemicellulose, protease, cellulose, α-amylase, alcalase, microbial mixed enzymes, etc.), supercritical water extraction, ultra-high pressure treatment, ultra-high electric field treatment, and ultrasound extraction (151, 152). These advanced methods exhibit a large handling capacity, lower processing time, higher purity, a mild condition, and an energy-efficient and environment-friendly way of operation (150). However, for efficient purification, proper precipitation is required, which was achieved through alcohol precipitation with washing (149). Growing, investigation proved that the purification of pectins is efficiently attained through stepwise innovative methods like ethanol precipitation, ultrafiltration in combination with diafiltration, ultrafiltration, and metal precipitation (153).

## Pharmaceutical Application of Microbial Pectinases and Derived Pectic Substances

In this modern era, various enzymes that are produced by microorganisms have been tremendously applied in different sectors of the industries that are directly or indirectly being used in day-to-day life. Enzymes are considered as the topmost biological tools due to their adjustable and specific catalytic properties owing to their increased applications in different biotechnological processes. Pectinases are one of the most significant industrial enzymes, and some of the notable contributions in pharmaceutical sectors are explained here.

### Dietary or Nutritive Fiber Preparation

Humans are omnivorous and regularly consuming diversified foods that need to be properly digested in the digestive system. On the other hand, friendly microbes present in the gastrointestinal tract comprehend convolution to digest them due to the entry of foreign complex compounds in a regular fashion. For which, fermentable dietary fibers through pectinase could be helpful in modulating the internal enzymatic system for proper digestion and to improve the immune system by acting as natural prebiotics. In certain pharmaceutical products, depolymerized pectins can be directly used to increase the viscosity and volume of stool and are also prescribed against acute diarrhea and constipation. Moreover, fermented pectic substances are used in throat lozenges as a decongestant in the form of a demulcent and also used in combination with certain products for the wound-healing process. The pectic elements are used as medicated adhesives against the treatment of colostomy. Pectins, with the combination of sulfate, are used to reduce the clotting time and can be applied in place of heparin. A complex made up of degraded pectin along with iron is helpful for the treatment of iron deficiency anemia (154–156).

### Oil Extraction

Essential oils from medicinal plants nowadays are widely adopted in both developed and developing countries due to their valuable medicinal properties against the treatment of different diseases including microbial infectious ailments, depression, anxiety, cancer, and wound healing with no or less side effects. Further, they are contributing a maximum role in cosmetics and perfume industries. However, the process of extraction through organic solvents might damage some important functional groups that are essential for healthcare benefits. To avoid this, most of the oil processing or pharmaceutical or cosmetic industry uses pectinase enzymes during the extraction process to destroy the emulsifying properties of pectin and promote the liquefaction of cell wall components, ultimately yielding a better volume of products. Moreover, the oils extracted through the enzymatic processes are enabled to retain the elevated concentration of phytocompounds like polyphenols, essential proteins, antioxidants, lipophilic bioactive compounds, and vitamin E with improving storage stability (157–160).

### Tablet Formulation

In tablet formulations, currently, pectin hydrogels are widely used as binding agents that act as a controlled releasing agent, which are mostly used in colon cancer treatments. Hydrolyzed pectin beads are formulated by adopting the ionotropic gelation method and are used as a sustained-release drug delivery system. The modulation of low-methoxy pectin by esterification or implementation of calcium pectinate gel beads in formulations also supports the administration of the drug. Basically, calcium pectinate is used because of the development of insoluble hydrophilic coatings which further interact with each other in the host cell and helps in the smooth release of targeted medicine (161, 162). Furthermore, a report also suggested the effectiveness and controlled release of oral formulations with compacting the pectic substances which were derived from orange and mango peels. These are having an excellent impact with functional polymers and have outstanding binding properties during the formulation of various tablets (163, 164).

### Formulation for Lowering Blood Glucose and Cholesterol

Direct pectin and/or pectic substances as obtained from pectinase treatment through the fermented process from the peels of various fruits and vegetables are being considered as a fruitful composition in pharma-based products due to their high fiber content. Scientists have reported and claimed that pectin is partially helping to prevent and treat severe diseases like diabetes and obesity, but it totally depends on the nature of viscosity, molecular weight, and degree of esterification. It is predicted that the more soluble fibers help to enhance gut viscosity, thus minimizing the re-absorption of bile acids. As a result, there is an increment of the synthesis of bile acids from cholesterol that helps the poor circulation of blood cholesterol. Herbal formulations related to this were tried in both *in vitro* and *in vivo* models by taking the digested pectins from the peels of apple, citrus, soybean etc. (132, 165, 166).

### Cosmetic Formulation

Currently, the pharmaceutical industry emphasizes on different cosmetic-related beauty products due to the most emerging demand by youths. The increasing environmental pollutants and unhealthy living practices have raised serious health impacts, including a detrimental impact on skin health. The pectic products are efficiently mixed with other chemicals for cosmetics as well as other personal care products which are found to be effective against various skin ailments. It is known not only for being a good emulsifier for thickening and gelling the face cream but also for its complex nature of polysaccharides that contain certain anti-oxidative and anti-aging properties that are able to prevent skin damage. However, the rate of efficacy may vary as per the sources from where it is extracted or fermented by respective pectinase enzymes (167–169).

## Conclusion

The microbial research has created an irreversible renaissance in the present era of innovations and cutting-edge research for finding the novel utility of microorganisms and their products, which is a sheer reality. It is concluded that the role of pectinases and/or pectins is found to be curiously recognizable in various industrial processes, with promising results. From the extensive research, it is clear that pectinolytic enzymes become the key imperative to innovate strategies for the significant development or improvement of enzymes relevant to industrial products. However, it is one of the key critical factors to ensure the cost viability for the production of these enzymes from selective microorganisms and implemented environmental conditions. As evidenced, the application of pectinase and derived pectic substances are least explored with respect to their application in pharmaceutical products. Therefore, it is urgently needed to strengthen and expand the usage of such valuable enzymes in the pharmaceutical industry. Further, the intervention of biotechnological aspects is critically needed for the development of a broad-spectrum pectinase with high catalytic affinities. Therefore, in-depth insights into a deeper understanding of the expression mechanism at the biochemical and molecular levels are essential.

## Author Contributions

SS and JR composed the first draft of the manuscript, whereas RK, HT, and SLS comprehensively edited the manuscript, which was then reviewed and edited by all authors.

## Conflict of Interest

The authors declare that the research was conducted in the absence of any commercial or financial relationships that could be construed as a potential conflict of interest.
